# The immediate global responses of *Aliivibrio salmonicida* to iron limitations

**DOI:** 10.1186/s12866-015-0342-7

**Published:** 2015-02-04

**Authors:** Sunniva Katharina Thode, Tim Kahlke, Espen Mikal Robertsen, Hilde Hansen, Peik Haugen

**Affiliations:** Department of Chemistry and The Norwegian Structural Biology Centre, Faculty of Science and Technology, UiT − The Arctic University of Norway, Tromsø, 9037 Norway; Current address: Environmental Genomics Team, CSIRO Marine and Atmospheric Research, Castray Esplanade, Hobart, 7000 TAS Australia

**Keywords:** *Aliivibrio salmonicida*, Iron homeostasis, Ferric uptake regulator, Siderophore, Bisucaberin, Microarray

## Abstract

**Background:**

Iron is an essential micronutrient for all living organisms, and virulence and sequestration of iron in pathogenic bacteria are believed to be correlated. As a defence mechanism, potential hosts therefore keep the level of free iron inside the body to a minimum. In general, iron metabolism is well studied for some bacteria (mostly human or animal pathogens). However, this area is still under-investigated for a number of important bacterial pathogens. *Aliivibrio salmonicida* is a fish pathogen, and previous studies of this bacterium have shown that production of siderophores is temperature regulated and dependent on low iron conditions. In this work we studied the immediate changes in transcription in response to a sudden decrease in iron levels in cultures of *A. salmonicida*. In addition, we compared our results to studies performed with *Vibrio cholerae* and *Vibrio vulnificus* using a pan-genomic approach.

**Results:**

Microarray technology was used to monitor global changes in transcriptional levels. Cultures of *A. salmonicida* were grown to mid log phase before the iron chelator 2,2’-dipyridyl was added and samples were collected after 15 minutes of growth. Using our statistical cut-off values, we retrieved thirty-two differentially expressed genes where the most up-regulated genes belong to an operon encoding proteins responsible for producing the siderophore bisucaberin. A subsequent pan-transcriptome analysis revealed that nine of the up-regulated genes from our dataset were also up-regulated in datasets from similar experiments using *V. cholerae* and *V. vulnificus*, thus indicating that these genes are involved in a shared strategy to mitigate low iron conditions.

**Conclusions:**

The present work highlights the effect of iron limitation on the gene regulatory network of the fish pathogen *A. salmonicida*, and provides insights into common and unique strategies of *Vibrionaceae* species to mitigate low iron conditions.

**Electronic supplementary material:**

The online version of this article (doi:10.1186/s12866-015-0342-7) contains supplementary material, which is available to authorized users.

## Background

Iron is an essential micronutrient for all living organisms [1-3], and withholding of iron is recognized as a first line of defence against microorganisms (e.g., bacteria) [4,5]. Extremely low iron concentrations create an efficient barrier against potential invading pathogens that may have entered the organism through, for example, an open wound on the skin surface. This defence strategy puts extraordinary pressure on invading pathogens to carry extremely efficient mechanisms to sequester iron from within the host [3,6]. Iron acquisition systems are therefore regarded as important virulence factors. Low iron conditions force pathogens into a stress mode, which results in the down-regulation of genes that encode iron-using and iron-storage proteins, and up-regulation of genes involved in iron acquisition [4,7,8]. Consequently, pathogenic bacteria often express and utilize multiple iron sequestering systems ranging from siderophore based systems, heme uptake systems and systems for uptake of free iron [8].

Although iron is an essential micronutrient, high concentrations of iron in the presence of oxygen are potentially harmful due to formation of oxidative radicals [9]; thus, influx and intracellular processing of iron must be tightly regulated. The ferric uptake regulator (Fur) is the main regulator of genes involved in iron uptake, storage and metabolism, and acts in an iron-dependent manner [10-13]. In *E. coli,* Fur acts mainly as a transcriptional repressor: at high iron concentrations it binds iron and forms homodimers which suppress the transcription of genes involved in a wide range of metabolic functions. Genes regulated by Fur not only include genes directly involved in iron homeostasis, but also DNA and energy metabolism, redox stress resistance, chemotaxis, bioluminscence and production of toxins and other virulence factors [2,10,14,15]. Fur is therefore regarded as a global regulator. Finally, it is also well established that Fur can indirectly activate gene expression by blocking the expression of the small RNA named RyhB, which typically targets mRNA for degradation. For example, RyhB targets the *fur* mRNA in a feedback regulation loop, and also targets mRNA that encode iron-using or iron-storing proteins like Bfr, SodB and FumA [16].

Fur recognizes and binds to a site on the DNA known as the Fur-box. Several alternative hypotheses for Fur-boxes have been proposed; for example a palindromic 19 bp site, three 6 bp repeats, and 7–17 motif [17-20]. In 2009, Ahmad and co-workers suggested a *Vibrio* Fur binding site consensus to be 5′-AATGATAATNATTTCATT-3′ [21]. This *Vibrio* consensus is similar to the suggested Fur box in other bacteria like *Bacillus subtilis*, *Yersinia pestis*, *E. coli* and *Pseudomonas aeruginosa* [22-25].

The importance of iron, and the elaborate regulation of iron uptake and homeostasis in bacterial cells in general, has prompted a number of researchers to study the roles of iron with regard to bacterial virulence and pathogenicity. In two recent studies, global responses to low iron conditions in cultures of *Vibrio vulnificus* and *Vibrio cholerae* (both human pathogens from the diverse family *Vibrionaceae*) were studied [11,26]. Here, cultures of *V. cholerae* and *V. vulnificus* were grown to mid log phase with iron chelators included in the growth medium from the beginning of the experiments. The results from these two experiments showed up-regulation of genes involved in siderophore biosynthesis and transport: TonB systems, heme transport and utilization, ferrous iron transport, and superoxide dismutase. In addition, the *V. vulnificus* experiment showed an up-regulation of a Tad-1 cluster.

We are studying the roles of iron in another *Vibrionaceae* representative, *Aliivibrio salmonicida. A. salmonicida* is the causative agent of cold-water vibriosis, and possesses several iron acquisition systems that may be important for its pathogenicity [27]. This assumption is based on the observation that the bacterium only produces significant amounts of siderophores when grown at or below 10°C [28,29], which coincides with the observation that outbreaks of cold-water vibriosis are normally associated with temperatures below 10°C [28]. Another intriguing feature of *A. salmonicida* is that it produces the dihydroxamate siderophore bisucaberin that has not yet been found in other *Vibrionaceae* representatives [30,31]. The bisucaberin biosynthesis genes (VSAL_I0134-I0136) in *A. salmonicida* strain LFI1238 are located on a genomic island that has likely been acquired by horizontal gene transfer from an unknown source [27]. The genome of the LFI1238 strain also harbors another siderophore biosynthesis system (VSAL_II0273-VSAL_II0278), which is commonly found in *Vibrionaceae*. However these latter genes are assumed to be disrupted and are annotated as pseudogenes [27]. Also, the transport of siderophores is carried out through siderophore receptors, and the energy for transport of the iron-siderophore complex across the membrane is provided by TonB systems. *Vibrionaceae* genomes usually contain 2–3 TonB systems [32-34]. The *A. salmonicida* genome encodes three TonB systems [27], where the TonB1 gene VSAL_I751 (*tonB1*) contains a frame-shift mutation and likely produce a non-functional protein.

Here, we have studied the immediate global responses in cultures of *A. salmonicida* to low iron conditions using microarray, and compared the results with comparable studies in *V. cholerae* [11] and *V. vulnificus* [26] using a pan-genome approach. In the two latter studies long-term responses to low iron was monitored (using microarray). We hypothesize that it is the immediate phase that is most critical for bacterial survival during iron starvation. Hence, we wanted to identify the genes that are first affected by low iron conditions, and avoid secondary effects such as unrelated stress responses. Our results provide new insights into how *A. salmonicida* responds to low iron conditions.

## Methods

### Bacterial strains, culture conditions and sampling

*A. salmonicida* strain LFI1238 [27] was cultured in LB medium containing 1% NaCl (Luria-Bertani broth Miller, Difco) at 8°C with 200 rpm in all experiments. To determine the optimal concentration of iron chelator 2,2'-dipyridyl (Sigma-Aldrich), *A. salmonicida* was grown to an optical density (600 nm) of 0.4 before the culture was split into 6 separate flasks. One flask was kept as control whereas 10–500 μM 2,2'-dipyridyl was added to the remaining cultures.

For Northern blots and microarray analysis (see below), six individual colonies (i.e. biological replicates) of *A. salmonicida* LFI1238 were grown until they reached an OD_600_ of approximately 0.5. The replicates were then split into two sub samples: one of these parallels was kept as the control, while 50 μM of the iron chelator 2,2’-dipyridyl was added to the other half. Samples were harvested after 15 min, spun down and frozen for later use.

### Total RNA purifications

For microarray analysis and Northern blotting total RNA was extracted from the cell pellets using IsolRNA (5prime) and DNA was removed using the DNA-*free* kit (Applied Biosystems). DNase-treated total RNA was subsequently run through RNeasy MinElute Cleanup columns (Qiagen) to remove any remaining contaminants, and to further concentrate the RNA. The RNA was finally dissolved in 16 μl RNase free water.

### Microarray analysis

cDNA was made by using the Amino Allyl Labeling cDNA Kit as described by the manufacturer protocol (Applied Biosystems). Reactions contained 18 μg total RNA. cDNA samples were labeled with the CyDye™ Post-Labelling Reactive Dye Pack (GE healthcare). Control samples (i.e. untreated samples) were labeled with Cy3, which produces green fluorescent light when scanned (at 532 nm), and treated samples were labeled with Cy5, which produces red light (at 635 nm). Two of the six slides were labeled in the opposite manner, and were used as dye-swap controls to adjust for unequal labeling efficiencies between the fluorescent dyes.

The labelled samples were hybridized to “*Vibrio salmonicida* V1.0.1 AROS” slides (Eurofins MWG Operon) at 42°C for 20 hours on a TECAN HS4800 hybridisation station, and microarray slides were subsequently washed, once in 0.1 × SSC/0.1% SDS for 5 min at 42°C, then once in 0.1 × SSC/0.1% SDS for 10 min at room temperature, and finally four times in 0.1 × SSC for 1 min at room temperature. Slides were scanned using a GenePix 4000D scanner (Axon Instruments Inc.) at 532 and 635 nm. Images were explored and initial data analyses were performed by using the GenePix Pro v6.1 software. The final analysis of expression data was done using the R-based Limma software.

### Northern blot analysis

Northern blot analysis was used to validate the microarray expression data. Treated and untreated RNA samples from each of the six biological replicates were pooled. Ten μg total RNA was separated on 1.2% denaturing formamide agarose gels, and run at 90 V for four hours in 1× MOPS buffer at 4°C. RNA was next transferred to a Hybond-N+ nylon membrane (Amersham) by capillary transfer. Selected gene specific dsDNA was amplified using PCR and labelled with [α-32P] and was used as probes according to the Amersham Megaprime DNA labeling system kit (GE healthcare). Hybridizations were performed over-night at 42°C using ULTRAhyb hybridization solution (Applied biosystems) and signals were acquired on phosphoimaging screens (Fujifilm) and scanned using a BAS-5000 phosphoimager (Fujifilm). Quantification of signals was done using the ImageGauge software v4.0 (Fujifilm) in profile mode. The intensities of the different bands were normalized to 16S rRNA probes.

### Computation of core, accessory and unique transcripts

To compare our microarray results with data from similar experiments using *V. vulnificus* strain CMCP6 [26] and *V. cholerae* strain 0395 [11], the protein sequences of differentially expressed genes were downloaded from GenBank (using geneID numbers). For *V. cholerae* the geneIDs are from the genome sequence of *V. cholerae* O1 N16961 and not strain O395 that was used in the experiment. Accession numbers for the *V. cholerae* O1 N16961 are AE003852 and AE003853, accession numbers for *V. vulnificus* CMCP6 genome are AE016795 and AE016796, and finally accession numbers for the *A. salmonicida* LFI1238 are FM178379 and FM178380. OrthoMCL [35] was used to identify core genes, i.e. genes present in all three genomes. Genes up-regulated in two datasets were denoted as accessory transcripts and unique transcripts were up-regulated in one dataset. Percent identity cut-off and percent match cut-offs were set to 50%. The inflation parameter was set to 0.

### Ethics statement

The research presented in this paper do not involve human subjects, and we see no ethical issues.

## Results and discussion

### Microarray analysis of iron depleted *A. salmonicida*

**Figure 1:**
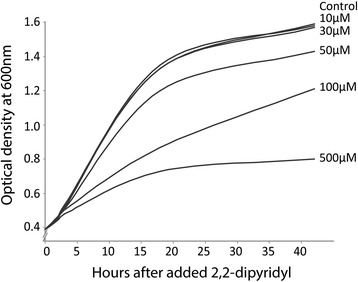
**Titration of 2,2’-dipyridyl concentration.** *A. salmonicida* strain LFI1238 was grown in LB containing 1% NaCl to an optical density at 0.4 (600 nm). The culture was split into six individual flasks and supplemented with different concentrations of 2,2'-dipyridyl before growth was monitored for 44 hours. Culture treated with 50 μM 2,2'–dipyridyl showed a slight reduction in growth and this concentration was therefore used in all subsequent experiments.

Using our model organism *A. salmonicida* we tested the immediate global changes in gene expression in response to low-iron stress conditions using a microarray approach. The iron chelator 2,2'-dipyridyl was used to create iron-limited conditions, and the appropriate chelator concentration was found by comparing the growth of *A. salmonicida* in the absence or presence of various 2,2'-dipyridyl concentrations. As shown in Figure 1, the growth of the bacterium was clearly affected when the growth medium contained 100 or 500 μM 2,2'-dipyridyl. Based on this result we decided to use 50 μM 2,2'-dipyridyl because it resulted in only a slight growth reduction, and we assumed that a strong growth inhibition would induce broader and less relevant stress responses.

Samples for microarray analysis were prepared by growing *A. salmonicida* in LB with 1% NaCl at 8°C. *A. salmonicida* requires NaCl for growth, and the NaCl concentration of the medium is known to affect growth, motility and other activities [36]. In our experiment we used a NaCl concentration close to the physiological conditions the bacterium would experience inside its natural host (Atlantic salmon) [37], as well as temperature where up-regulation of iron sequestration systems is known to occur, and the bacterium is known to develop cold water vibriosis [28,29]. The cultures were grown to mid log phase (OD_600nm_ 0.5) before 2,2’-dipyridyl was added to a final concentration of 50 μM, and samples were collected after 15 minutes to monitor immediate responses.

Table 1 lists 32 differentially expressed genes that fulfilled our criteria (fold change values ≥ 1.5 and p-values ≤ 0.10). These threshold values were chosen after evaluating alternative combinations of cut-off values, and evaluating the biological soundness of the resulting data (i.e. keep maximum valuable data while minimizing the introduction of noise). Also, biological replicates tend to create more variation between samples compared to technical replicates, and too strict cut-off values can therefore exclude biologically sound data. In our analysis, all differentially expressed genes (Table 1) are up-regulated (in treated sample) and associated with predicted Fur-boxes [21]. Moreover, based on the current annotations, the majority (at least 21 of 32) of genes have predicted functions in iron homeostasis. The operon associated with the highest fold change values contains three genes (*bibABC*) for biosynthesis of the siderophore bisucaberin. Interestingly, of all sequenced bacteria in the relatively large *Vibrionaceae* family, *A. salmonicida* was until recently the only representative with this system. Using the amino acid sequences for the *bibABC* genes in a Blast search we identified homologous genes (99% identity) in the very closely related *Aliivibrio logei* [38]. This observation favours that the system was acquired by horizontal gene transfer in the most recent common ancestor of the two *Aliivibrio* species. This scenario is more parsimonious than the alternative, which is that the system was lost in all other *Vibrionaceae* representatives. Other genes on the list with functions in iron metabolism include siderophore receptors, heme receptors and the associated ATP-binding cassette (ABC) and TonB transport systems. The gene encoding ferrioxamine B receptor (BfrH) is possibly encoding a siderophore receptor (ferrioxamines are siderohores). The same operon encodes a TonB3 system. The operon encoding FhuC and FhuD (associated with siderophore-iron transport) is also up-regulated under iron limited conditions. The CDSs encoding TolR2, a TonB dependent receptor and a putatively exported protein are located in the same operon and are all up-regulated. A recent publication suggests that TolR is likely TtpC, which is necessary for stabilisation of TonB2 binding in *Vibrio anguillarum* and *V. cholerae* [39]. TonB1 is the only TonB, which appears to be up-regulated. Apparently, this TonB is most likely non-functional due to a frameshift in *A. salmonicida*.

**Table 1:** **Differentially expressed genes in** ***A. salmonicida*** **LFI1238 stimulated with 50** μ**M 2,2'-dipyridyl**

**CDS**	**Gene**	**Product** ^**1**^	**Fold change** ^**2**^	**p-value** ^**2**^
***Transport/binding proteins***				
VSAL_I1734		heme receptor (pseudogene)	1.5	0.03
VSAL_I1751	*tonB1*	TonB protein (pseudogene)	5.0	0
VSAL_I1752	*exbB1*	TonB system transport protein	2.4	0.01
VSAL_I1754	*hmuT*	heme transporter protein, putative periplasmic binding protein	4.3	0
VSAL_I2257	*feoA*	ferrous iron transport protein FeoA	1.8	0.06
VSAL_I2258	*feoB*	ferrous iron transport protein FeoB	1.8	0.07
VSAL_I2259	*feoC*	ferrous iron transport protein FeoC	1.8	0
VSAL_I2588	*fbpA*	iron(III) ABC transporter, periplasmic iron-compound-binding protein	2.1	0.08
VSAL_II0110		TonB dependent receptor	3.4	0
VSAL_II0112	*tolR2*	biopolymer transport protein	2.0	0
VSAL_II0150	*fhuC*	ferrichrome transport ATP-binding protein	3.2	0
VSAL_II0151	*fhuD*	ferrichrome-binding periplasmic protein	3.2	0.01
VSAL_II0909	*bfrH*	ferrioxamine B receptor	3.3	0
VSAL_p320_27		iron ion ABC transporter, periplasmic component	2.4	0.01
VSAL_p320_29		iron ion ABC transporter ATP-binding protein	1.7	0.07
***Adaptation***				
VSAL_I1749	*huvX*	heme uptake and utilization protein	1.7	0
***Biosynthesis of cofactors, carriers***				
VSAL_I0134	bibA*	Bisucaberin siderophore biosynthesis protein A	7.6	0
VSAL_I0135	bibB*	Bisucaberin siderophore biosynthesis protein B	5.8	0.01
VSAL_I0136	bibC*	Bisucaberin siderophore biosynthesis protein C	1.9	0.06
VSAL_I1750	*phuW*	putative coproporphyrinogen oxidase	2.2	0
***Cell envelope***				
VSAL_I1248		membrane protein	2.9	0
VSAL_I1785		putative exported protein	2.2	0
VSAL_I1786		putative iron-regulated protein	2.8	0
VSAL_I1864		putative outer membrane protein	4.2	0
VSAL_II0074		membrane protein	3.4	0
VSAL_II0111		putative exported protein	2.3	0
VSAL_II0717		putative membrane protein	1.6	0.02
VSAL_II0868		putative lipoprotein	3.4	0
***sRNA***				
VSAL_I3102s		VSsrna22 small RNA RyhB	4.6	0
***Unknown function, no known homologues***				
VSAL_I2980		hypothetical protein	1.5	0.1
VSAL_I2892		hypothetical protein	3.7	0
VSAL_II0148		hypothetical protein	3.8	0

^1^Annotated product of CDS ^2^Fold change values are shown for ≥ 1.5 differentially expressed genes with p-values ≤ 0.1. Positive fold change value indicate up-regulation compared to untreated control. ^*^ *bibA* is annotated as L-2,4-diaminobutyrate decarboxylase in the *A. salmonicida* genome annotations, and *bibB and bibC* are annotated as iucD and iucC, respectively [26,30].

None of the differentially expressed genes on our lists were down-regulated (i.e. did not fulfil the cut-off criteria). This is surprising since *ryhB* is moderately up-regulated (4.6 fold) under low iron conditions, and down-regulation of known RyhB targets is expected based on evidence from other species. There are two possible explanations for this finding: the data is valid and all significantly differentially expressed genes are up-regulated, however we cannot rule out that unknown technical issues have affected our data leading to this result. Although we have not validated any potential RyhB targets by Northern blot analysis, the overall agreement between fold change values in our microarray and Northern blot data (see below) support the conclusion that the microarray data is valid and we have no reason to suspect serious technical issues. Known RyhB targets that are identified in both *E. coli* and *V. cholerae* include *sodB*, *sdhC*, *fumA* and *gltB1* [40,41]. All these are present in our dataset, but are not differentially expressed (fold change −1.03, −1.16, −1.10 and −1.27, respectively). In *V. cholerae,* 31 genes are up-regulated in a *ryhB* null mutant; however, the fold change values for these potential RyhB targets are very moderate (majority varies between 1.6 − 3.3 fold) [41]. Similarly, when RyhB is over-expressed in *E. coli,* fold change values for the majority of down-regulated genes vary between 1.5 − 6 [40]. Based on this information, it may not be surprising that secondary effects such as for example RyhB regulation is not detected in our experiment considering that; i) RyhB is only moderately up-regulated, ii) *A. salmonicida* has a relatively long doubling time (6–8 hours) at 8°C, and iii) we measured effects after a very short exposure time (15 min) to the iron chelator.

### Northern blot analysis

**Figure 2:**
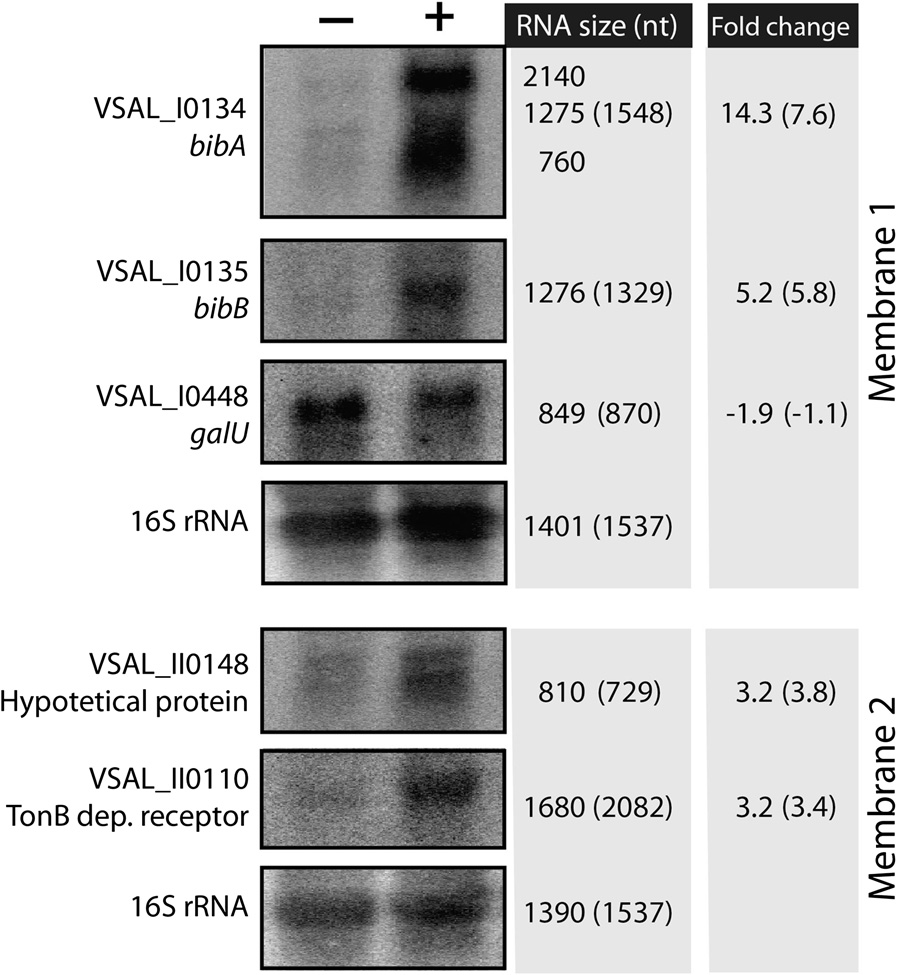
**Validation of selected microarray results using Northern blot analysis.** RNAs from six replicates of *A. salmonicida* were pooled and separated on denaturating 1.2% formamide agarose gels, transferred to two membranes and tested for presence of specific RNAs using radio-labeled probes. Plus (+) indicates addition of 50 μM of the iron chelator 2,2'-dipyridyl to the cultures 15 min prior to sampling, whereas minus (−) indicates the untreated control. Numbers in the left column indicate the size of the RNA as measured from the gel, and numbers in parentheses indicate the theoretical size. The right column indicate normalized fold change values calculated from the Northern blot autoradiograms, while numbers in parentheses show the corresponding microarray fold change values.

Northern blot analysis was used to validate the microarray expression data of 5 up-regulated genes; VSAL_I0134, VSAL_I0135, VSAL_I0448, VSAL_II0148, VSAL_II0110. The intensities of the different bands were normalized to 16S rRNA. Autoradiogram pictures are shown in Figure 2, and show that the Northern blot data are in good overall agreement with the microarray analysis. For example, for VSAL_I0135 (*bibB*) the microarray and Northern blot analyses show fold change values of 5.8 and 5.2, respectively, and for VSAL_II0110 the respectively fold change values were 4.5 and 3.2. The microarray fold change value that differs most in magnitude from the Northern Blot result is for VSAL_I0134 (*bibA*). Here, the microarray and Northern Blot values were 7.6 and 14.7, respectively.

### Comparison of results with other global expression profiling studies from *Vibrionaceae*

Next, we wanted to compare our result to similar global expression profiling studies (microarray) where the response of other representatives of the *Vibrionaceae* family to low-iron conditions was studied. By uncovering responses that are shared between bacteria belonging to the *Vibrionaceae* family*,* or that are unique to one species, we may eventually provide a deeper understanding of mechanisms involved in virulence. Two such datasets are currently available: Crosa and co-workers [26] tested responses of *V. vulnificus* strain CMCP6 to iron-limiting conditions by adding 50 μM ethylenediamine-di-(o-hydroxyphenylacetic) acid (EDDA) (iron-depleted conditions) to TSBS medium cultures from the beginning, in addition to untreated controls, and harvested cells at mid-log phase (i.e. OD_600nm_ 0.6-0.8). Three biological replicates were pooled before cDNA synthesis to avoid culture variations in microarray analysis. In their analysis they were able to identify 49 genes that are differentially up-regulated during iron-depleted conditions. In another study by Mey et al. [11], *V. cholerae* strain 0395 was grown in EZ RDM defined medium with no added iron (i.e. iron-depleted conditions), or EZ RDM with 40 μM of ferrous sulfate (iron-replete conditions) to OD_650nm_ 0.3. In their study, they identified 84 differentially expressed genes during iron-depleted conditions.

**Figure 3:**
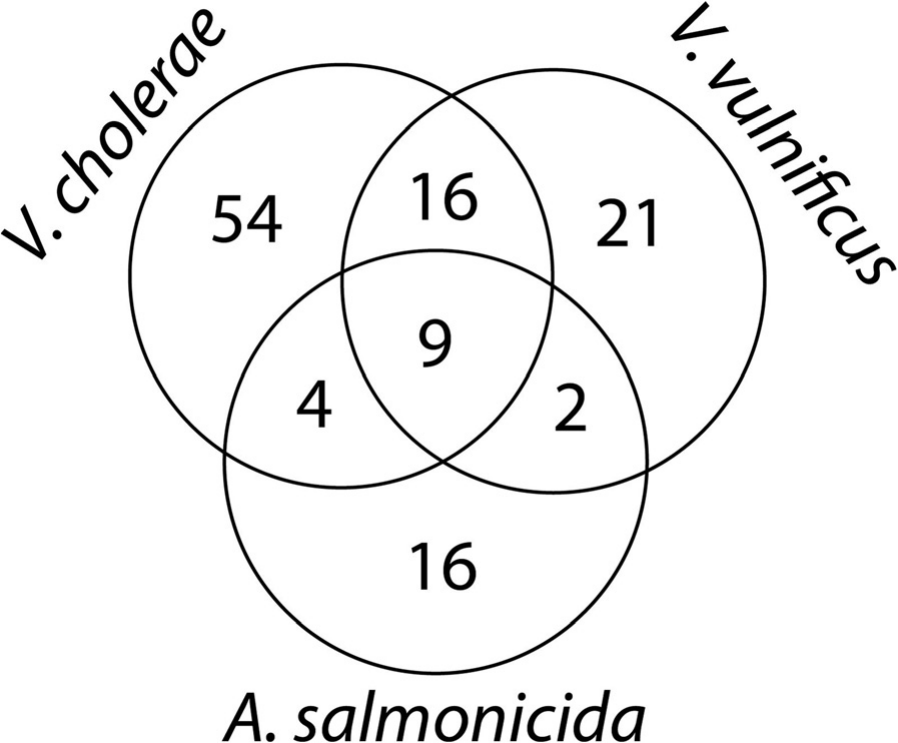
**Venn diagram summarizing numbers of transcripts that are differentially expressed in** ***A. salmonicida*** **,** ***V. cholerae*** **and** ***V. vulnificus*** **under low-iron conditions.** Numbers are based on this study, and the microarray studies using *V. cholerae* [11] and *V. vulnificus* [26].

In our comparative analysis we adopted the Pan genome concept and organized the differentially-expressed transcripts into core, accessory and unique transcripts. A Venn-diagram representation of the comparative analysis are shown in Figure 3. More detailed information about the comparative analysis results are found in Additional file 1. Core transcripts are differentially expressed in all datasets, unique transcripts are differentially expressed in one dataset, whereas accessory transcripts are differentially expressed in two datasets. Although the three experiments were performed differently (e.g., different growth media, different iron chelators/iron-deplete medium, different sampling time/cell densities, etc.), we believe they could identify potentially interesting common or unique responses to low iron conditions among the three bacteria.

We used the software OrthoMCL [35] with percent identity and percent match cut-off set to 50 and the inflation value set to 0 to identify potential homologs. Subsequently, we curated the generated homology clusters manually and identified 9 core transcripts (10 in *V. vulnificus* as VV1_1660 and VV1_1661 are paralogs, and both cluster together with VC0608 in *V. cholerae* and VSAL_I2588 in *A. salmonicida*). Three of the core transcripts belonging to a ferrous iron transport system (*feoA, feoB* and *feoC*), one transcript belonging to a TonB system (*exbB1*), two transcripts encode proteins that are potentially involved in heme uptake/utilization, two transcripts which may belong to a TonB2 system, and finally one transcript encoding a ferric iron ABC transporter periplasmic iron-compound-binding protein. Therefore, all differentially expressed core transcripts encode proteins involved in iron homeostasis.

Sixteen accessory transcripts are shared between *V. vulnificus* and *V. cholerae*. These encode products involved in siderophore biosynthesis, siderophore and heme transport and utilization, iron storage (*bfd* and *bfr*), and oxidative stress response (*sodA*). Of the 16 accessory genes shared between *V. cholerae* and *V. vulnificus,* ten are not present in the *A. salmonicida* genome (e.g. *bfr*, *bfd* and the different *vtc* component genes). Moreover, the finding that three siderophore biosynthesis sequences (VC0771/VV2_0838, VC0773/VV2_0835 and VC0774/VV2_0834) are shared only between *V. vulnificus* and *V. cholerae* does not seem reasonable, and may reflect the fact that some siderophore biosynthesis proteins are more related between *V. vulnificus* and *V. cholerae* than they are to the bisucaberin biosynthesis system in *A. salmonicida*. Four accessory transcripts are shared between *V. cholerae* and *A. salmonicida* (VSAL_I2892/VC0091, VSAL_I1786/VC1264, VSAL_I1785/VC1265 and VSAL_II0074/VC1588), and finally two accessory transcripts are shared between *A. salmonicida* and *V. vulnificus* (i.e. TonB1 VSAL_I1751 and VV21614, and a periplasmic heme binding protein encoded by VSAL_I1754 and VV21611). The fact that *V. cholerae* and *V. vulnificus* share the highest number (i.e. sixteen) of common up-regulated transcripts is reasonable since they are more closely related to each other than to *A. salmonicida*. In addition, the experimental conditions used for *V. cholerae* and *V. vulnificus* are more similar.

Fifty-four, twenty-one and sixteen transcripts are unique to *V. cholerae, V. vulnificus* and *A. salmonicida*, respectively, and of these at least eighteen, eight and ten transcripts are directly associated with iron homeostasis. In *V. cholerae* the unique transcripts encode proteins with functions in vibriobactin biosynthesis (*vibD-H*), siderophore transport (*viuA, viuC, viuD, viuG, viuP, irgA, vctA, fhuA* and *fhuC*), heme transport (*hutA* and *hutD*), iron transport (*tonB1, tonB2* and *exbD2*), transcription regulation (*irgB* and *vctR*), various enzymatic catalysis (e.g. *ligA-2*, *fumC*, *ptrB*, *napA-D*, *napF*, *menB*), and finally hypothetical functions. In *V. vulnificus* half of the eight unique transcripts encode proteins that are involved in vulnibactin biosynthesis and transport (VV20839, VV20840, VV20841 and VV20844), and the remaining four have functions in iron transport, i.e. TonB systems (VV21618, VV20359 and VV20360) and an ABC-type Fe^3+^ transport protein (VV11662). In *A. salmonicida* unique transcripts are directly associated with bisucaberin biosynthesis genes (*bibA*, *bibB* and *bibC*), ferrioxamine B receptor (*bfrH*), ferrichrome binding (*fhuC* and *fhuD*), iron transport, i.e. an ABC transport system (VSAL_p320_27 and VSAL_p320_29), a TonB2 dependent receptor (VSAL_II0110), and a heme receptor (VSAL_I1734).

### Siderophore biosynthesis in *Vibrionaceae*

The approximately 150 different *Vibrionaceae* species (157 species in the NCBI taxonomy database when excluding unclassified sp. and subspecies) [42] have the potential to synthesize various siderophore iron chelators. For example, *V. cholerae* encodes the system *VibABCDEFH* that is responsible for the production of vibriobactin. Similarly, *A. salmonicida* contains the *bibABC* genes, which encode enzymes involved in production of the bisucaberin siderophore [30]. *V. vulnificus* produces the species specific siderophore vulnibactin [43]. Vulnibactin is structurally similar to vibriobactin, but its biosynthesis pathway is not fully understood [44]. The genes *venB*, *vvsA* and *vvsB* are involved in the biosynthesis, but their roles are unclear. *V. vulnificus* also synthesizes a hydroxamate-type siderophore. Unfortunately, neither its structure nor its biosynthetic pathway have been identified [45].

In our analysis the three genes involved in bisucaberin biosynthesis in *A. salmonicida* top our list of differentially up-regulated genes/operons. The result resembles expression data from both *V. cholerae* [11] and *V. vulnificus* [26] where siderophore biosynthesis genes were highly up-regulated (although they did not top the list of up-regulated genes) after being grown in low iron medium. Together these results strongly support the idea that siderophore production and utilization represent one of the first and probably most important responses to mitigate low iron conditions. It is however still unclear why different *Vibrio/Aliivibrio* species use different siderophores. One possible explanation is that the utilization of multiple siderophores represents an advantage in the competition for scarce resources. However, some vibrios can partly mitigate such strategies by utilizing siderophores produced by other bacteria.

### TonB systems

In Gram negative bacteria the transport of ferri-siderophores and heme across the membrane requires energy. The energy is provided by TonB systems, which consist of the TonB, ExbB and ExbD proteins. In vibrios TonB2 systems also include the TtpC protein [1,39]. Vibrio genomes typically contain two or three TonB systems [32,46]. Interestingly, in our analysis *tonB1*, *tonB2* and *exbD2* from *V. cholerae,* and *tonB2* from *V. vulnificus,* are considered unique, whereas the remaining TonB transcripts (*exbB1*, *exbB2* and *exbD1*) are either core or accessory transcripts. *tonB1* is shared between *A. salmonicida* and *V. vulnificus* even though the *A. salmonicida tonB1* gene is a pseudogene (contains one frameshift mutation). *tonB2* transcripts were not identified in the *A. salmonicida* microarray dataset. Intriguingly the *V. cholerae tonB1* transcript was considered unique, but after further examinations we realized that this transcript was excluded from the results because it was just below the cut-off settings for identities, while the TonB1 transcripts from *V. vulnificus* and *A. salmonicida* were just above the cut-off setting. This show the weaknesses of a small dataset and the problems of setting specific cut-offs.

## Conclusions

We studied the immediate effect of low iron conditions, and compared this to similar studies where effects were examined after prolonged growth in low iron conditions. We identified 32 up-regulated genes, whereas no genes were found to be down-regulated. Although caution should be taken in extrapolating *in vitro* results to what may occur *in vivo*, it is our belief that studies such as those performed here will provide a better understanding of iron uptake and metabolism in bacteria, and eventually provide us with some insights into their virulence and survival mechanisms, their ability to adapt to changing environmental conditions, and finally their evolution. We have studied expression of genes that are essential for iron homeostasis in a single species, but by studying a collection of species from a broader spectrum of bacteria e.g., from the same family (i.e. *Vibrionaceae*), unique and common strategies for mitigating low iron conditions can be identified. A future goal for us is to use such knowledge to compare environmental isolates with known pathogens to better understand the relevance of iron homeostasis in virulence. Finally, increased knowledge on iron uptake systems and regulation is highly relevant to on-going efforts where such systems are used as targets for potential drugs with the goal to control pathogenic bacteria.

### Availability of supporting data

Microarray data are available in the ArrayExpress database under accession number GSE57996.

## Additional file

Additional file 1: Table S1. The table lists differentially expressed (up-regulated) core, accessory and unique transcripts in *A. salmonicida*, *V. vulnificus* and *V. cholerae*. Amino acid sequences of corresponding genes were retrieved from ENA, and used as input for clustering of orthologs using OrthoMCL with amino acid percent match and percent identity cut-offs set to 50%.

## Abbreviations

PCR: Polymerase chain reaction
LB: Luria Bertani broth
Fur: Ferric uptake regulator
dsDNA: Double-stranded DNA
rpm: Rounds per minute
OD: Optical density
min: minutes
MOPS: 3-(N-morpholino)propanesulfonic acid
SSC: Saline-sodium citrate
SDS: Sodium dodecyl sulfate
CDS: Coding sequence
rRNA: Ribosomal RNA
mRNA: Messenger RNA
sRNA: Small RNA
*AS*: *Aliivibrio salmonicida*
ABC: ATP-binding cassette
